# Forced Vital Capacity and Low Frequency Reactance Area Measurements Are Associated with Asthma Control and Exacerbations

**DOI:** 10.1007/s00408-022-00542-1

**Published:** 2022-06-03

**Authors:** Rory Chan, Brian Lipworth

**Affiliations:** Scottish Centre for Respiratory Research, School of Medicine, Ninewells Hospital, University of Dundee, Dundee, DD1 9SY Scotland UK

**Keywords:** Air trapping, Forced vital capacity, Oscillometry, Small airways, Asthma control, Exacerbations, Reactance area

## Abstract

**Introduction:**

Forced vital capacity (FVC) is often preserved in severe asthma unless there is evidence of either airway remodelling or air trapping. Area under the reactance curve (AX) can be used to assess small airways dysfunction related lung stiffness and is related to disease control in severe asthma.

**Methods:**

We explore if there may be a potential synergistic interaction between FVC and AX in terms of impaired asthma control as ACQ and exacerbations requiring oral corticosteroids (OCS). We pragmatically defined < 100% and ≥ 1.0 kPa/L/s as impaired FVC or AX, respectively.

**Results:**

Patients with combined impairment of FVC and AX had significantly worse asthma control as higher ACQ, more severe exacerbations requiring OCS and worse spirometry (FEV_1_ and FEF_25–75_) than those with impaired FVC but preserved AX.

**Conclusion:**

This in turn supports using both spirometry and oscillometry to characterise airway physiology more comprehensively in patients with more severe asthma.

## Introduction

Forced vital capacity (FVC) is often preserved in severe asthma unless there is evidence of either airway remodelling or air trapping [1]. In this regard, as residual volume increases, FVC declines in commensurate fashion. In turn reduced FVC in severe asthma may also reflect reduced lung compliance [2]. Air trapping and airway remodelling are hallmarks of small airways dysfunction (SAD) in severe asthma [3]. The presence of SAD can also be assessed by oscillometry which is an effort independent test for frequency-dependent impedance. In particular, area under the reactance curve (as AX) at 5 Hz and the resonant frequency can be used to assess SAD related lung stiffness and is related to disease control in severe asthma [4].

Here we explore if there may be a potential synergistic interaction between FVC and AX in terms of impaired asthma control as ACQ and exacerbations requiring oral corticosteroids (OCS), whilst also looking at the relationship to type 2 (T2) biomarkers as peripheral blood eosinophils, FeNO and total IgE.

## Methods

Data from 181 moderate-to-severe asthma patients with paired spirometry and oscillometry measurements were retrospectively collected from the National Health Service Tayside health informatics database. Spirometry (Micromedical, Chatham, UK) was performed according to ERS/ATS guidelines. Oscillometry was measured using either Masterscreen (Carefusion Hoechberg, Germany) or Tremoflo (Thorasys, Montreal, Canada). Measurements were performed in triplicate to assess oscillometry according to the ERS technical standards with oscillometry always performed prior to spirometry. We chose pragmatic arbritary cut points of < 100% and ≥ 1.0 kPa/L to denote impairment of FVC and AX, respectively. FeNO was measured using NIOX VERO (Circassia, Oxford, UK) according to the manufacturer’s instructions and ATS guidelines. Blood testing was performed for peripheral blood eosinophils and total IgE. Asthma control was determined using the 6-point asthma control questionnaire (ACQ), and the number of OCS-requiring asthma exacerbations in the preceding year was noted.

Statistical analysis was performed using SPSS version 27. Data were assessed for outliers and for normality with Shapiro–Wilks prior to analysis. An overall analysis of variance was performed to evaluate any significant differences in spirometry (mean 95% CI) between the four groups followed by pairwise comparisons with Bonferroni correction and a two tailed alpha error set at 0.05. Significant comparisons for oscillometry, T2 biomarkers, ACQ and OCS exacerbations (median, IQR) were performed using Mann–Whitney *U* tests. For National Health Service patients, Caldicott approval was obtained whilst for clinical trial patients informed consent and ethical approval was obtained via the East of Scotland research ethics service prior to data collection. In patients receiving biologic therapy, measurements were obtained prior to treatment initiation.

## Results

Mean demographic data comprised: gender (F/M), 115/66; age, 51 years; ICS beclomethasone equivalent dose, 1641 µg/day; ex-smokers, 17%; BMI, 31 kg/m^2^; FEV_1_, 84%; FVC, 101%; LABA, 83%; LAMA, 46%; LTRA, 52%; THEO, 19%; OAH, 49%; anti-IL5(rα), 18% and anti-IL4rα, 3%.

Patients with combined impairment of FVC and AX had significantly worse asthma control as higher ACQ, more severe exacerbations requiring OCS and worse spirometry (FEV_1_ and FEF_25–75_) than those with impaired FVC but preserved AX (Table 1). No significant differences in T2 biomarkers were observed. A similar pattern was detected in patients with preserved FVC and impaired AX versus those with preservation of both FVC and AX except there were no differences in exacerbations (Table 1).

**Table 1:** Significant differences in asthma control, exacerbations, pulmonary function and T2 biomarkers comparing FVC ≥ 100%, AX < 1.0 kPa/L versus FVC ≥ 100%, AX ≥ 1.0 kPa/L; and FVC < 100%, AX < 1.0 kPa/L versus FVC < 100%, AX ≥ 1.0 kPa/L

	FVC ≥ 100%		FVC < 100%	
	AX < 1.0 kPa/L	AX ≥ 1.0 kPa/L	AX < 1.0 kPa/L	AX ≥ 1.0 kPa/L
Age	49.4 (45.5–53.4) (*n* = 52)	53.6 (48.8–58.4) (*n* = 33)	45.4 (39.4–51.3) (*n* = 22)	54.9 (51.3–58.6)** (*n* = 49)
BMI (kg/m^2^)	28.7 (27.2–30.3) (*n* = 53)	32.7 (30.4–35.0)** (*n* = 26)	30.1 (27.9–32.3) (*n* = 27)	33.2 (31.2–35.2) (*n* = 41)
ACQ	1.8 (1.7) (*n* = 52)	2.2 (2.3)* (*n* = 33)	2.0 (2.8) (*n* = 22)	2.6 (1.6)* (*n* = 49)
OCS exacerbations	1 (3) (*n* = 53)	1 (4) (*n* = 26)	1 (4) (*n* = 27)	4 (3)* (n = 41)
FEV_1_ (L)	3.02 (2.84–3.20) (*n* = 62)	2.33 (2.06–2.59)*** (*n* = 35)	2.50 (2.27–2.73) (*n* = 30)	1.80 (1.61–1.98)*** (*n* = 54)
FEF_25–75_ (L/s)	2.44 (2.14–2.73) (*n* = 62)	1.46 (1.21–1.71)*** (*n* = 35)	1.95 (1.52–2.37) (*n* = 30)	1.24 (1.02–1.46)** (*n* = 54)
FVC (L)	4.19 (3.95–4.42) (*n* = 62)	3.48 (3.10–3.86)** (*n* = 35)	3.52 (3.30–3.75) (*n* = 30)	2.77 (2.55–2.98)*** (*n* = 54)
FEV_1_/FVC	73.2 (70.3–76.0) (*n* = 62)	66.4 (63.1–69.7)** (*n* = 35)	71.2 (66.4–76.0) (*n* = 30)	65.5 (61.5–69.5) (*n* = 54)
R5-R20 (kPa/L/s)	0.06 (0.05) (*n* = 62)	0.16 (0.10)*** (*n* = 35)	0.08 (0.05) (*n* = 30)	0.28 (0.25)*** (*n* = 54)
X5 (kPa/L/s)	− 0.12 (0.07) (*n* = 52)	− 0.25 (0.10)*** (*n* = 28)	− 0.11 (0.06) (*n* = 23)	− 0.37 (0.33)*** (*n* = 49)
AX (kPa/L)	0.45 (0.49) (*n* = 62)	1.84 (1.42)*** (*n* = 35)	0.44 (0.44) (*n* = 30)	3.31 (3.85)*** (*n* = 54)
FeNO (ppb)	24 (27) (*n* = 52)	24 (19) (*n* = 31)	22 (24) (*n* = 21)	25 (31) (*n* = 44)
PBE (cells/µL)	300 (240) (*n* = 59)	300 (313) (*n* = 33)	392 (296) (*n* = 26)	200 (305) (*n* = 49)
Total IgE (kU/L)	118 (255) (*n* = 49)	72 (381) (*n* = 27)	157 (365) (*n* = 26)	160 (374) (*n* = 42)

*p < 0.05 **p < 0.01 ***p < 0.001, denotes Bonferroni corrected comparisons for spirometry between groups for either FVC ≥ 100% or FVC < 100% Values presented as median (IQR) except for spirometry, age and BMI where means (95%CI) were used

## Discussion

We have shown that asthma patients with coupled impairment of FVC and AX have worse clinical outcomes than those with impaired FVC but preserved AX. It is worth noting that the median difference in ACQ score was 0.6-units exceeding the minimally clinically important difference of 0.5. Moreover, it has traditionally been accepted that ACQ is a good predictor of future risk of severe exacerbations thereby placing these findings into relevant clinical context [5].

Patients with preserved FVC and impaired AX had worse spirometry as FEV_1_ and FEF_25–75_ than those with preserved FVC and AX. For FEV_1_, the mean difference amounted to 690 ml which would be considered as being a clinically relevant difference. Hence clinicians might conceivably be lulled into a false sense of security in an individual with preserved FVC by using spirometry alone. From a practical point of view oscillometry is much easier to perform during normal tidal breathing and is more physiological than the artificial expiratory manoeuvre with spirometry. We acknowledge that the ratio of residual volume to total lung capacity is a better method to assess air trapping, albeit unlikely to be performed on a routine basis in a busy real-life clinic.

Our data show the greatest difference in ACQ and exacerbations occurred in those with combined impairment of AX and FVC. This in turn points to using both spirometry and oscillometry to characterise airway physiology more comprehensively in patients with moderate to severe asthma.

## Abbreviations

ACQ: Asthma control questionnaire
ATS: American Thoracic Society
AX: Area under reactance curve
ERS: European Respiratory Society
FEF: Forced expiratory flow rate between 25 and 75% of forced vital capacity
FeNO: Fractional exhaled nitric oxide
FEV_1_: Forced expiratory volume in 1 s
FVC: Forced vital capacity
ICS: Inhaled corticosteroids
MCID: Minimal clinically important difference
OCS: Oral corticosteroids
R5-R20: Difference in resistance between 5 and 20 Hz
SAD: Small airways dysfunction
T2: Type 2
